# Paraventricular Nucleus of the Thalamus Neurons That Project to the Nucleus Accumbens Show Enhanced c‐Fos Expression During Early‐Stage Cue‐Reward Associative Learning in Male Rats

**DOI:** 10.1111/ejn.70168

**Published:** 2025-06-21

**Authors:** S. Seeger‐Armbruster, M. Wang, R. E. Campbell, B. I. Hyland

**Affiliations:** ^1^ Department of Physiology School of Biomedical Sciences University of Otago Dunedin New Zealand; ^2^ Brain Health Research Centre University of Otago Dunedin New Zealand; ^3^ Centre for Neuroendocrinology University of Otago Dunedin New Zealand

**Keywords:** immediate early gene, midline thalamus, retrograde adeno‐associated viral vector

## Abstract

The paraventricular nucleus of the thalamus (PVT) is a central node in brain networks controlling motivated behaviors. It processes inputs from cerebral cortex, brainstem, and hypothalamus and has efferents that project to a wide range of structures, including the nucleus accumbens (nAcc). It is known that PVT neurons projecting to the nAcc show c‐Fos activation in response to reward‐related cues, in well‐trained animals. We previously found that c‐Fos expression is also increased early in the conditioning process, during the first session of learning a new cue‐reward association in rats, but neurons with projections to nAcc were not identified in that study. Here, we tested the hypothesis that nAcc‐projecting PVT neurons would show this enhanced c‐Fos expression following first exposure to the association of a visual cue with a subsequent food reward. Male rats were stereotaxically injected in the nAcc with a retrogradely transported adeno‐associated virus construct leading to green fluorescent protein (GFP) expression in cell bodies of afferents from PVT. Following a single session of cue‐reward training, processing of the brains with dual immunohistochemistry for c‐Fos and GFP showed significantly higher density of double labelled neurons in the conditioned group, compared to controls in which the same number of cues and rewards were delivered at random intervals with respect to each other. Such activation of immediate early gene expression in PVT to nAcc projecting neurons very early in paired associative reward learning may have a role in modulating plasticity in the nAcc.

## Graphical abstract

Retrogradely transported AAV was injected bilaterally into nAcc, leading to GFP expression in PVT projection neurons. In the control group, a visual cue and food rewards occurred at random intervals. In the S→R group, the cue preceded the reward by a fixed interval. After one task session, double immunohistochemistry for GFP and c‐Fos expression revealed significantly increased density of c‐Fos positive nuclei in PVT projection neurons in the S→R group compared to controls. Created with BioRender.com.

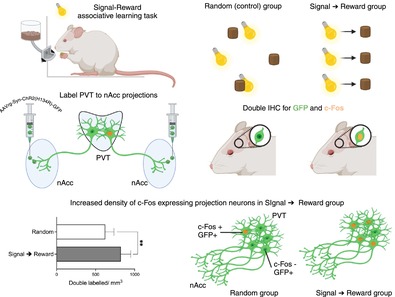

**Abbreviations**
AAV: adeno‐associated viral vector
ANOVA: analysis of variance
AP: anteroposterior
C: chamber only (experimental group label)
CL: chamber + light (experimental group label)
CR: chamber + reward (experimental group label)
DAPI: 4′,6‐diamidino‐2‐phenylindole
GFP: green fluorescent protein
nAcc: nucleus accumbens
PBS: phosphate‐buffered saline
PVA: anterior region of paraventricular nucleus of the thalamus
PV: mid region of paraventricular nucleus of the thalamus
PVP: posterior region of paraventricular nucleus of the thalamus
PVT: paraventricular nucleus of the thalamus
SD: standard deviation
SEM: standard error of the mean
S‐R: signaled reward (experimental group label)

## 1 Introduction

The paraventricular nucleus of the thalamus (PVT) is a midline nucleus with a critical role in motivated behaviors such as arousal, feeding, and addiction (Kirouac 2015; Millan et al. 2017; Vertes et al. 2022). For instance, it is engaged in regulating pavlovian conditioned sign‐ or goal‐tracking behavior (Haight and Flagel 2014), in computing risk–reward comparisons (Worden et al. 2021), in feeding control (Kelley et al. 2005; Ye et al. 2022), in modulating stress and anxiety (Hsu et al. 2014; Kirouac 2021), and also in reward processing and drug addiction behavior (Millan et al. 2017; Zhou and Zhu 2019; De Groote and de Kerchove d'Exaerde 2021). These diverse correlates are consistent with a global role in integrating information about prior learning with competing needs and current internal state to guide appropriate behavior for restoring or defending homeostasis (Penzo and Gao 2021).

These functions reflect the extensive connectivity of PVT. Inputs to the nucleus arise from cerebral cortex, and numerous sites in the brain stem and hypothalamus with diverse functional roles (Van der Werf et al. 2002; Li and Kirouac 2012; Vertes et al. 2022). The results of the integration within PVT of information from these afferents is in turn distributed to several target structures via output neurons that are uniformly excitatory across the whole nucleus (Kirouac 2025). These targets include more ventral nuclei of the midline thalamus, prelimbic, infralimbic and subicular cortex, various hypothalamic nuclei, the amygdala, the striatum, and the nucleus accumbens (nAcc) (Van der Werf et al. 2002; Kirouac 2025). Of these outputs, the numerically largest projection is to the nAcc (Su and Bentivoglio 1990; Moga et al. 1995; Pinto et al. 2003; Dong et al. 2017), a key node controlling reward‐related behavior (Ikemoto and Panksepp 1999). In the nAcc, PVT axons synapse directly on medium spiny neurons, and also on dopaminergic fibers (Pinto et al. 2003; Ligorio et al. 2009), enabling complex modulation of both dopamine signaling within the nucleus (Jones et al. 1989; Parsons et al. 2007) and activity of nAcc output neurons (Kirouac 2015).

These input and output connections lead to the hypothesis that the PVT integrates information regarding internal states, which have strong alerting and arousal properties, with cortical processing related to the emotional salience of external cues, and transmits the outcome of this integration to the nAcc for the context‐specific modulation of behavior (Kirouac 2015; Millan et al. 2017; De Groote and de Kerchove d'Exaerde 2021; Iglesias and Flagel 2021; Penzo and Gao 2021; Vertes et al. 2022). A prediction of this hypothesis therefore is that a population of PVT neurons should respond to salient sensory cues, and that this property should be seen in PVT neurons projecting to nAcc.

Both reward and punishment invest associated signals with enhanced salience. Accordingly, many studies using a range of methods including calcium imaging, c‐Fos staining, and single neuron recording have found positive associations with reward‐related factors in PVT neurons (Brown et al. 1992; Dayas et al. 2008; Choi et al. 2010; Igelstrom et al. 2010; Flagel et al. 2011; Yager et al. 2015; Li et al. 2016; Zhu et al. 2018; Choi et al. 2019; Munkhzaya et al. 2020). However, others found c‐Fos activation in single neurons by reward stimuli only in the specific subpopulation of PVT neurons projecting to nAcc, not in the population as a whole (Hamlin et al. 2009; Haight et al. 2017), and in one of these, the effect was further restricted to only the posterior subregion of PVT (Haight et al. 2017). Further, a calcium imaging study found that reward‐association induced an *inhibitory* response in this nAcc‐projecting PVT subpopulation (Otis et al. 2019).

There are several possible reasons for such variant findings between studies, as noted in a recent review by De Groote and de Kerchove d'Exaerde (2021). For example, different methods for assessing neural activation differ in sensitivity, specificity, temporal resolution, and whether they measure responses at the population or single‐neuron level. Studies also utilize a wide range of different behavioral paradigms which may engage PVT differently. Furthermore, studies vary in the stage of learning at which animals are tested. Thus, while we and others have investigated early stages of learning (e.g., Igelstrom et al. 2010; Choi et al. 2019; Otis et al. 2019), others have measured activity following an extinction procedure (e.g., Flagel et al. 2011; Yager et al. 2015; Haight et al. 2017), or in response to reinstatement following extinction (e.g., Hamlin et al. 2009). Finally, there are also potential differences in subpopulations of PVT neurons according to their anteroposterior location in the nucleus, in the dominant sources of afferent inputs, predominant efferent projection targets, and neurotransmitter receptor profiles (Barson et al. 2020; Kirouac 2025). Such differences may result in different neuronal response patterns to reward stimuli (e.g., Haight et al. 2017; Choi et al. 2019), which could lead to differences between studies if different regions were investigated.

In previous work, we found enhanced c‐Fos labeling in an unidentified population of PVT neurons immediately following the first session in which animals were exposed to a cue‐reward pairing, using a specific associative‐learning paradigm (Igelstrom et al. 2010). In that study, we only sampled a single anteroposterior level and did not identify the subpopulation of PVT neurons projecting to nAcc. Here, we investigated whether the subpopulation of PVT neurons that project to the nAcc also shows enhanced activation. Given the potential for paradigm and methodological specificity and for anteroposterior gradients across the nucleus, we used the same behavioral paradigm as the previous study but sampled and compared measures for anterior, mid, and posterior regions of PVT. The results confirmed that in this paradigm, nAcc‐projecting PVT neurons across the entire PVT show elevated c‐Fos expression following first exposure to a cue‐reward pairing.

## 2 Materials and Methods

### 2.1 Animals and Surgical Procedures

All experimental work was approved by the University of Otago Animal Ethics Committee, and in accordance with the National Institute of Health Guide for the Care and Use of Laboratory Animals (NIH Publications No. 80‐23, revised 1996), all efforts were made to minimize the number of animals used and their suffering. Adult male Wistar rats (*n* = 31; 257–321 g at beginning of the experiments) were group housed in environmentally controlled conditions in a reversed 12‐h light/dark cycle. All experiments were conducted during the rats' dark phase. There were two separate experiments: a baseline study (*n* = 15 rats) and a main study (*n* = 16 rats).

### 2.2 Behavioral Apparatus

Behavioral sessions were carried out in an experimental chamber made of clear acrylic (floor area 22.5 × 16 cm^2^) with a feeding trough protruding from the wall and a house‐light above, in a quiet, darkened room. A pellet dispenser (ENV‐ 203IR, Med Associates, United States) delivered food rewards through a tube into the feeding trough, with the activation of the dispenser emitting an audible click. Nose pokes into the trough were detected by interruption of an infrared beam passing across the trough. A house‐light located above the chamber was used as a cue in some experimental groups. It illuminated the whole chamber from above and was visible to the rat independent of its position in the chamber. Activations of the dispenser and light cues, and processing of infrared beam interruptions were controlled by a custom script in Med‐PC IV (Med Associates, United States; RRID:SCR_012156), which also produced outputs marking the numbers of trials, rewards, light cues, nose pokes, and the first nose poke latency following reward delivery for each reward trial. These data were imported into Excel for offline analysis.

### 2.3 Baseline Experiment Procedures

Many stimuli can activate c‐Fos expression in neurons, which could affect baseline levels and thus the ability to detect subtle effects between task‐variant groups. We assessed this by measuring the baseline level of c‐Fos expression in rats exposed to different components of the experimental context, in the absence of any co‐presentation of cues and rewards. For this, rats (*n* = 15) were first habituated to handling for a total of 7 days, and then on the next day were put in the chamber for the experimental session, which lasted 30 min. The rats were divided into three groups (*n* = 5 each). One group was exposed to the chamber alone, with all the associated handling and transport, with no lights or food reward delivered, referred to as group C. The second group was exposed to the chamber, with the addition of random light‐cue flashes but no food delivery (group CL). The third group was exposed to the chamber plus food reward delivery (with associated auditory, gustatory, and rewarding stimuli), without any light cues (group CR). Rats were removed from the chamber immediately after the session and placed in individual cages in a ventilated holding cabinet for 30 min followed by histological processing described further below. Prior to behavioral testing, all rats in all groups underwent stereotaxic surgery to inject a retrograde adeno‐associated viral vector (AAV) expressing green fluorescent protein (GFP) bilaterally, targeting the nAcc, using the same methods as described below for the main experiment. However, across the three groups, there were insufficient numbers with successful bilateral injections for meaningful statistical analysis of GFP labeled cells, and so the data are not presented here.

### 2.4 Main Experiment Surgical Procedures

**FIGURE 1:**
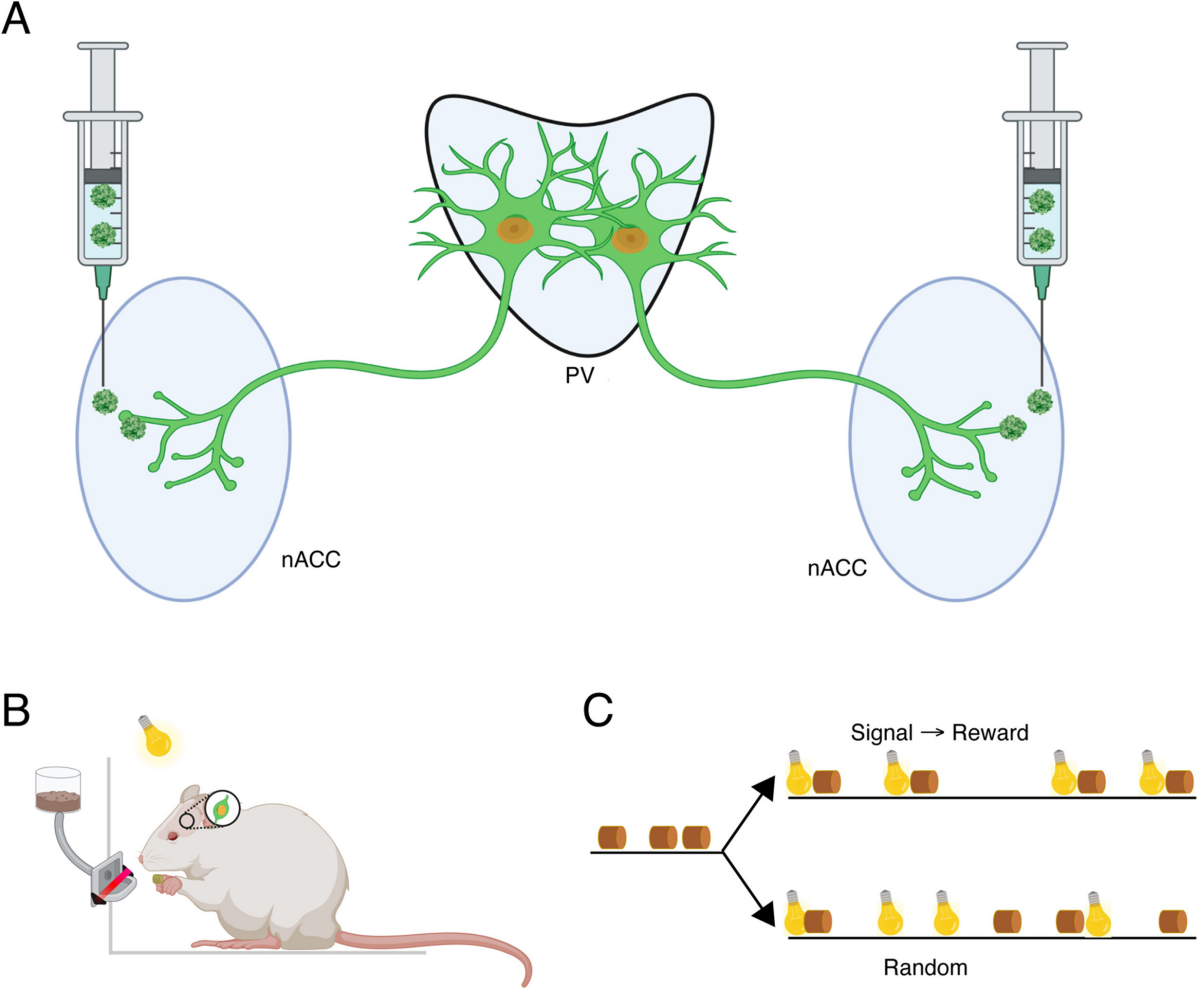
Experimental overview and design. (A) Bilateral injections of AAVrg‐Syn‐ChR2(H134R)‐GFP (green particles) into nucleus accumbens (nAcc) led to GFP expression (green) in paraventricular nucleus (PVT) neurons. Neurons were labelled for nuclear c‐Fos expression (orange). (B) In the baseline and main experiments, animals were exposed to various behavioral situations that could include delivery of food pellets (brown cylinder), light cues (yellow bulb), or no particular events. Food pellet retrieval was detected by infra‐red beam interruption (red bar across feeder entrance). (C) Behavioral situations for the main experiment. Pretraining involved random delivery of food pellet rewards. Rats were then split into two groups; in the signal→reward group, food delivery was preceded by a light cue at a constant interval, whereas in the random (control) group, food and lights were delivered at random times relative to each other. Created with BioRender.com.

Rats used for the main experiment (*n* = 16) underwent stereotaxic surgery to inject a retrograde AAV expressing GFP into the nAcc, to achieve expression of GFP in the cell membrane of nAcc‐projecting neurons in PVT (Figure 1A). Viral vector injections were performed under aseptic conditions and antibiotic cover (amphoprim, 30 mg/kg; Virbac Animal Health). Rats were deeply anaesthetized with isoflurane (initial 4%, continued with 2%; Attane, Bayer Animal Health NZ), placed in a stereotaxic frame (flat skull position) and injected s.c. along the intended incision line with the long‐acting local anesthetic marcaine (0.1 mL; 0.5% Bupivacaine, Claris Life Sciences Limited). The skull was exposed by a sagittal incision, and holes were drilled above the nAcc on each side. Injections of 0.2 μL (at 0.1 μL/min) AAVrg‐Syn‐ChR2(H134R)‐GFP construct (genomic titer: 5 × 10e13 gc/mL; Addgene viral prep #58880AAVrg) were made into the nAcc using a micro syringe (33 G cannula; World Precision Instruments). A ChR2 construct was used with a view to potential optogenetic experiments, but these were not pursued in this cohort of animals. The target coordinates were − 6.6 mm below the brain surface at anteroposterior (AP) + 2.2 mm and mediolateral ± 0.8 mm (relative to bregma) (Paxinos and Watson 2009). The cannula remained in place for 10 min after the infusion stopped before being slowly withdrawn. The incision was sutured, the rat was injected s.c. with 5 mL of 0.9% saline, and the long‐acting analgesic carprofen (5 mg/kg; Carprive, Norbrook). The rat was removed from the stereotaxic frame and placed in a warmed recovery cage. Post‐surgery animals were single‐housed and checked twice daily for the first week of recovery and then returned to group housing.

### 2.5 Main Experiment Behavior Procedures

After 1 week recovery from surgery, rats were habituated to handling and to the chamber in the experimental room during daily 35‐min sessions for 2 weeks. During the second of these, they were put on a restricted diet (18 g/day standard chow) and a mix of banana, grape, and chocolate flavored dustless Precision Pellets (45 mg, Bio‐Serv, United States) were placed on the chamber floor at the beginning of each session to familiarize them with the reward food. The following week, rats were exposed to 5 days of pre‐training sessions (35 min) during which rats learned to retrieve the sugar pellets from the trough (Figure 1B). For this, the dispenser was activated at pseudorandom intervals of 10–20 s in 0.25 s steps, with this interval beginning at the first nose poke after the release of the previous reward. Because the duration of the time in the chamber was fixed during pre‐training, the number of rewards retrieved provided a marker of familiarization with the task requirements and a threshold for successful learning was set to ≥ 2 consecutive sessions with more than 70 rewards retrieved per session.

In week 5 post‐surgery, rats underwent a single experimental session. Rats were divided into two groups, referred to as “signaled reward” (S‐R) and “random.” Both groups were exposed to the house‐light cue for the first time, along with the previously experienced food delivery. The key experimental difference between the groups was the temporal relationship between the cue and the reward delivery (Figure 1C). In the S‐R group (*n* = 8), release of the reward occurred immediately at the end of each 2 s illumination of the light (i.e., a “delay‐conditioning” procedure). Following the first nose poke after the release of the previous reward, there was an additional pseudorandom period of 10–20 s in 0.25 s steps before the delivery of the next cue‐reward pair. In contrast to the pre‐training sessions, which were only time limited, this experimental session continued until either 100 rewards were retrieved or 30 min had passed, whichever occurred first. For the random group (*n* = 8), the arrangements were the same except that light cue illumination and reward delivery were completely independent of each other in time. The reward delivery algorithm was as for the pre‐training. Following reward delivery, the system waited until the first subsequent nose poke and then generated an additional random interval of 10–20 s. The intervals between reward deliveries were thus at least 10 s, with variations in the animal's behavioral response latency generating an additional random delay. In this condition, the light activation sequence was controlled by a completely independent algorithm, which triggered another light stimulus at pseudorandom intervals of 10–20 s following the end of the previous light. This design is the same as we previously used to study PVT responses to cue‐reward pairing (Igelstrom et al. 2010). Rats were removed from the chamber immediately after the session and placed in individual cages in a ventilated holding cabinet for 30 min prior to histological processing.

### 2.6 Immunohistochemistry

Thirty minutes after the end of the experimental session, rats were deeply anaesthetized with pentobarbital (100 mg/kg, intraperitoneal; Provet) and transcardially perfused with 300 mL of cryoprotectant (10% sucrose, BioFroxx) followed by 300 mL of 4% paraformaldehyde (Merck) in 0.1 M phosphate buffer (pH 7.3; Merck). Brains were extracted from the skull 2–3 h after the perfusion, allowing for some firming of the tissue by the fixative to facilitate removal, and then post‐fixed in 4% paraformaldehyde overnight followed by immersion in 30% sucrose for at least 48 h for additional cryoprotection. Brains were coronally sectioned (40 μm) using a freezing microtome (Leica SM2400) and slices containing nAcc (2.5–0.6 mm anterior of bregma) and PVT (0.84–4.08 mm posterior of bregma; Paxinos and Watson 2009) were stored in cryoprotectant at − 20 °C.

Tissue selection for immunohistochemistry processing from each rat included 4–6 nAcc slices spanning the injection coordinates and 17–21 PVT slices spanning the entire AP extent of the nucleus, including anterior (PVA), mid (PV), and posterior (PVP) regions as defined by Paxinos and Watson (2009), in both cases with selected slices 160 μm apart (every 4th slice from the original serial sections). The tissue was processed in batches with each batch including tissue from rats from all groups in the experiment as well as a negative control that was not incubated in primary antibody. All sections coming out of cryoprotectant were initially washed in phosphate buffered saline (PBS; 3 × 10 min).

PVT slices were processed to visualize c‐Fos protein and GFP. A sequential staining protocol was used to amplify the c‐Fos signal. For this, endogenous peroxidases were blocked by incubation in 0.9% H_2_O_2_ (40% methanol/PBS) for 10 min at room temperature, followed by 3× PBS washes and incubation in primary antibody rabbit anti‐c‐Fos (1:5000; Abcam Cat# ab190289, RRID:AB_2737414) in incubation solution (PBS/0.3% Triton X‐100 [Sigma]/0.25% bovine serum albumin [BSA; GIBCO] + 2% normal goat serum [NGS; GIBCO]) for 48 h at 4 °C. After 4× PBS washes, slices were incubated in biotinylated goat anti‐rabbit secondary antibody (1:200; Vector Laboratories Cat# BA‐1000, RRID:AB_2313606) in incubation solution for 1 h at room temperature, followed by 4× PBS washes. Then, slices were incubated in VECTASTAIN Elite ABC‐Peroxidase Kit (1:100; Vector Laboratories Cat# PK‐6100, RRID:AB_2336819) in incubation solution for 45 min at room temperature, followed by 4× PBS washes, and incubation in biotinylated tyramide (1:333; Biotin‐XX Tyramide Reagent, Thermo Fisher Scientific Cat# B40951) in PBS with 0.0015% H_2_O_2_ for 20 min at room temperature. After another 4× PBS washes, slices were incubated in Streptavidin Alexa Fluor 568 conjugate (1:400; Thermo Fisher Scientific Cat# S‐11226, RRID:AB_2315774) secondary antibody in incubation solution for 2 h at 37 °C, washed 4×, and then incubated in chicken anti‐GFP (1:5000; Aves Labs Cat# GFP‐1020, RRID:AB_10000240) primary antibody in incubation solution + 2% NGS for 48 h at 4 °C. Following 3× PBS washes, slices were incubated in goat anti‐chicken AlexaFluor488 (1:500; Abcam #ab150173, RRID:AB_2827653) secondary antibody in incubation solution for 2 h at room temperature. After this incubation with secondary antibody, all sections went through a series of PBS and PB washes and were then mounted on charged glass slides (SuperfrostPlus) and allowed to dry for ~30 min. Finally, slides were coverslipped with Fluoromount‐G (with 4′,6‐diamidino‐2‐phenylindole (DAPI); Invitrogen #E119437) as a mounting medium.

NAcc slices were processed for single‐label GFP staining, with incubation in blocking solution (incubation solution: PBS/0.3% Triton X‐100 (Sigma)/0.25% bovine serum albumin (BSA; GIBCO); + 5% normal goat serum (NGS; GIBCO)) for 30 min at room temperature, after which the tissue followed the above protocol from the stage of the incubation in chicken anti‐GFP primary antibody.

### 2.7 Image Analysis

The extent of GFP labelling in nAcc and staining for GFP‐labelled cell bodies and c‐Fos‐labelled nuclei in PVT was visualized with a fluorescence microscope (Nikon Ti2‐E, Otago Micro and Nanoscale Imaging Unit). Overview images showing the whole slice were taken from all sections of nAcc and PVT and used to delineate the boundaries of the respective structure in Inkscape 0.92.4 (RRID:SCR_014479) with the aid of a stereotaxic rat atlas (Paxinos and Watson 2009).

Z‐stack images (steps = 2 μm) containing all three microscopy channels were taken from a single 10x objective frame showing the PVT from each slice and analyzed in ImageJ (NIH, 1.52a; RRID:SCR_003070). For this, each z‐stack was cropped to the outline of the PVT as obtained from the overview image and atlas overlay, the area of the PVT was measured, and the z‐stack was then converted into a single image plane using the Max Intensity projection.

For analysis of c‐Fos, the c‐Fos color channel was converted into an 8‐bit image to allow setting of the threshold level to include positive nuclei, which was applied consistently across all images, followed by quantification using the built‐in ImageJ Particle Analysis command. Then, for counting GFP‐labelled cells and GFP‐c‐Fos double labelled cells, all color channels were merged into one image, and neurons were counted using the Multi‐point tool. Because we used a ChR2 construct, GFP was localized to neuronal membranes. The GFP labelled cells were manually identified by scanning across all layers of the z‐stacks. Only cells with a clearly demarcated green labelled membrane around the cell body that encompassed a DAPI labelled nucleus were included in the count. Each of the three PVT subregions was quantified. Because the regions differ in volume, nuclear counts were normalized by conversion to densities (count/mm^3^) for statistical analysis.

### 2.8 Statistical Analysis

Data are given as mean ± standard deviation (SD) in text. Figures showing grouped data analyzed with analysis of variance (ANOVA) use box and whisker plots (mean, median, 25th to 75th percentiles, all data points and minimum and maximum values). Graphs of data pooled across factors show mean + standard error of the mean (SEM). All statistical analyses were performed with PRISM (GraphPad; RRID:SCR_002798). Exact *p* values are reported except where *p* < 0.001. Contrasts involving multiple factors or levels of factors were performed with two‐ or one‐way ANOVA followed by Holm‐Šidák post hoc tests. Pairwise contrasts involving only two groups were performed using the Mann–Whitney *U* test and correlation between two measures was assessed using non‐parametric Spearman *r*. Confirmation of potential outlier values was done using Grubbs' test with alpha = 0.0001. Contingency analyses used Fisher's exact test.

## 3 Results

### 3.1 Baseline c‐Fos Expression

The expression of c‐Fos is affected by a wide range of events, including the basic components of any associative learning task, such as handling, and novel stimuli or environments (Tischmeyer and Grimm 1999). To establish a baseline of c‐Fos expression in PVT relative to the various subcomponents of the main experiment, we quantified c‐Fos expression in a separate experiment in which groups of rats were exposed to different components of the experimental context, in the absence of any co‐presentation of cues and rewards. As described in the methods, group C was exposed to the chamber alone, with all the associated handling and transport, with no lights or food reward delivered, group CL was exposed to the chamber, with the addition of random light‐cue flashes but no food delivery, and group CR was exposed to the chamber plus food reward delivery (with associated auditory, gustatory, and rewarding stimuli), without any light cues. Because in the CR group, food pellets were only delivered following retrieval of a previous pellet, there could be variation between animals in the number of reward pellets obtained in the time‐limited session, depending on their attention to reward retrieval. Across the five rats, this ranged from 8 to 111 (median 88) retrievals, while average c‐Fos densities across all regions ranged from 5.62 × 10^3^ to 18.355 × 10^3^ nuclei/mm^3^. However, there was no significant correlation between the density of c‐Fos positive neurons and the number of rewards retrieved (*r*_*s*_ = −0.400, *p* = 0.517, Spearman correlation). Therefore, data from all rats in the group were used for the intergroup comparison.

**FIGURE 2:**
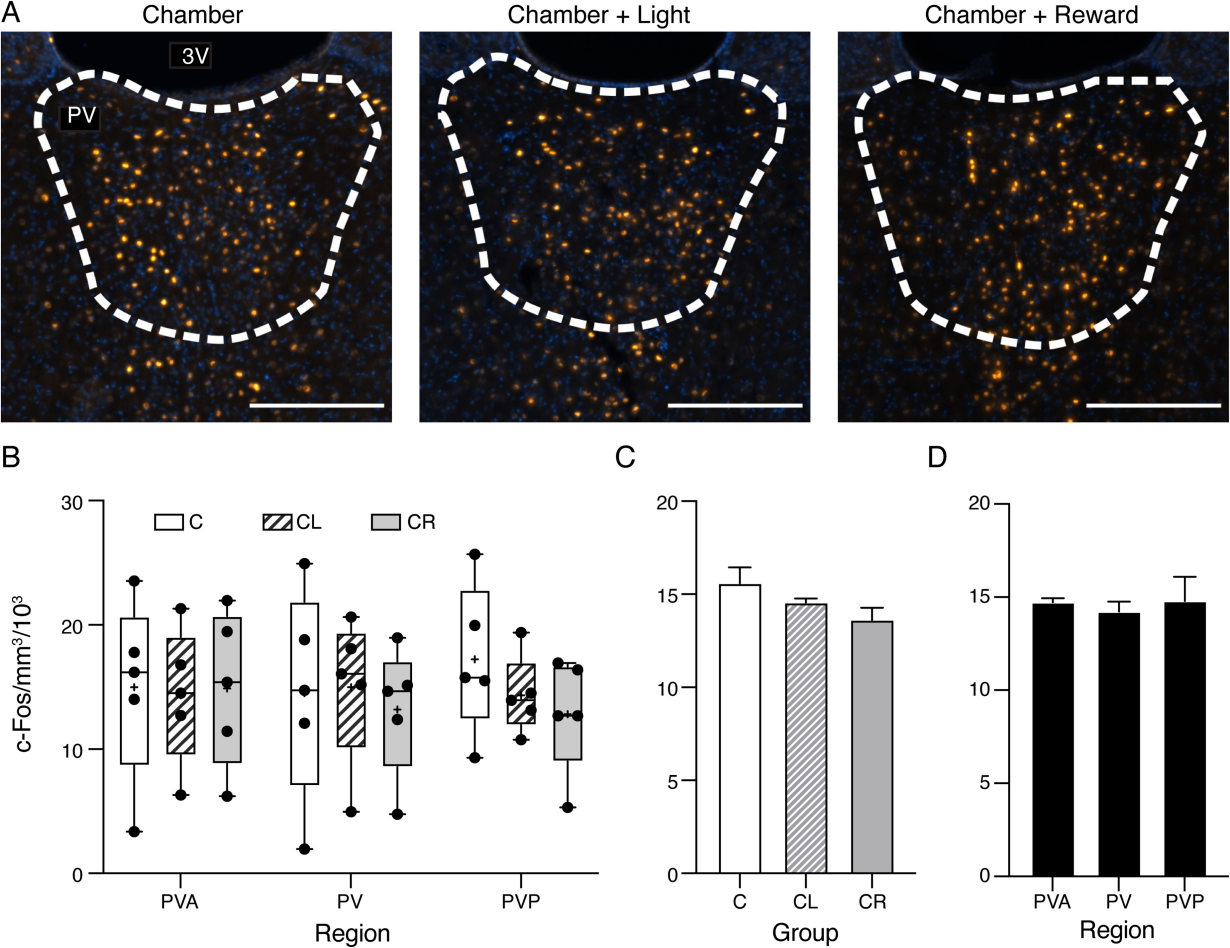
c‐Fos positive nuclei in the baseline experiment. (A) Histological transverse sections at the level of mid PVT (AP ‐2.4; PV) from three different rats showing typical nuclear staining for c‐Fos (orange) following exposure to each of the three behavioral contexts. Scale bars 250 μm. DAPI staining in blue. 3 V, third ventricle. For this illustration only (not for analysis), c‐Fos staining appearance has been enhanced by assigning “hot orange” to the channel, instead of orange. (B) Box and whiskers plot shows all data points (black dots), median (horizontal line) and mean (+ symbol) density of c‐Fos labelled nuclei (in thousands/mm^3^) for rats in chamber only (C, white bars), chamber + light (CL, striped bars), and chamber + reward (CR, grey bars) groups, split by PVT region (PVA, anterior; PV, mid; PVP, posterior). Box shows 25th to 75th percentiles, and whiskers mark minimum and maximum values. Two‐way ANOVA revealed no significant main effects or interaction. (C) Graph shows mean ± SEM density of c‐Fos nuclei for the three groups, pooled across PVT regions. (D) Graph shows mean ± SEM c‐Fos nuclei density for the three PVT regions, pooled across groups.

The results of the experiment are shown in Figure 2. The typical example sections shown in Figure 2A suggest no obvious differences in c‐Fos staining across the three groups. This was confirmed by the quantitative analysis (Figure 2B) with two‐way ANOVA for the factors *Group* (C, CL, CR) and *Region* (PVA, PV, PVP) finding no significant main effects of *Group* (F_(2, 36)_ = 0.390, *p* = 0.680), or *Region* (F_(2, 36)_ = 0.040, *p* = 0.961), and no interaction (F_(4, 36)_ = 0.220, *p* = 0.926). Pooled across regions, the mean ± SD density was 15.576 ± 1.461 × 10^3^, 14.553 ± 0.374 × 10^3^ and 13.626 ± 1.111 × 10^3^ nuclei/mm^3^ for C, CL and CR groups, respectively (Figure 2C). Pooled across groups (Figure 2D), the mean ± SD density was 14.731 ± 0.353 × 10^3^, 14.225 ± 0.934 × 10^3^, and 14.798 ± 2.251 × 10^3^ nuclei/mm^3^ for PVA, PV, and PVP, respectively. Thus, in the absence of any explicit cue‐reward conditioning, exposure to the experimental environment generated a consistent baseline density of c‐Fos positive nuclei of around 14–15 × 10^3^ nuclei/mm^3^ across the whole PVT.

### 3.2 Main Experiment

#### 3.2.1 Behavioral Analysis

**FIGURE 3:**
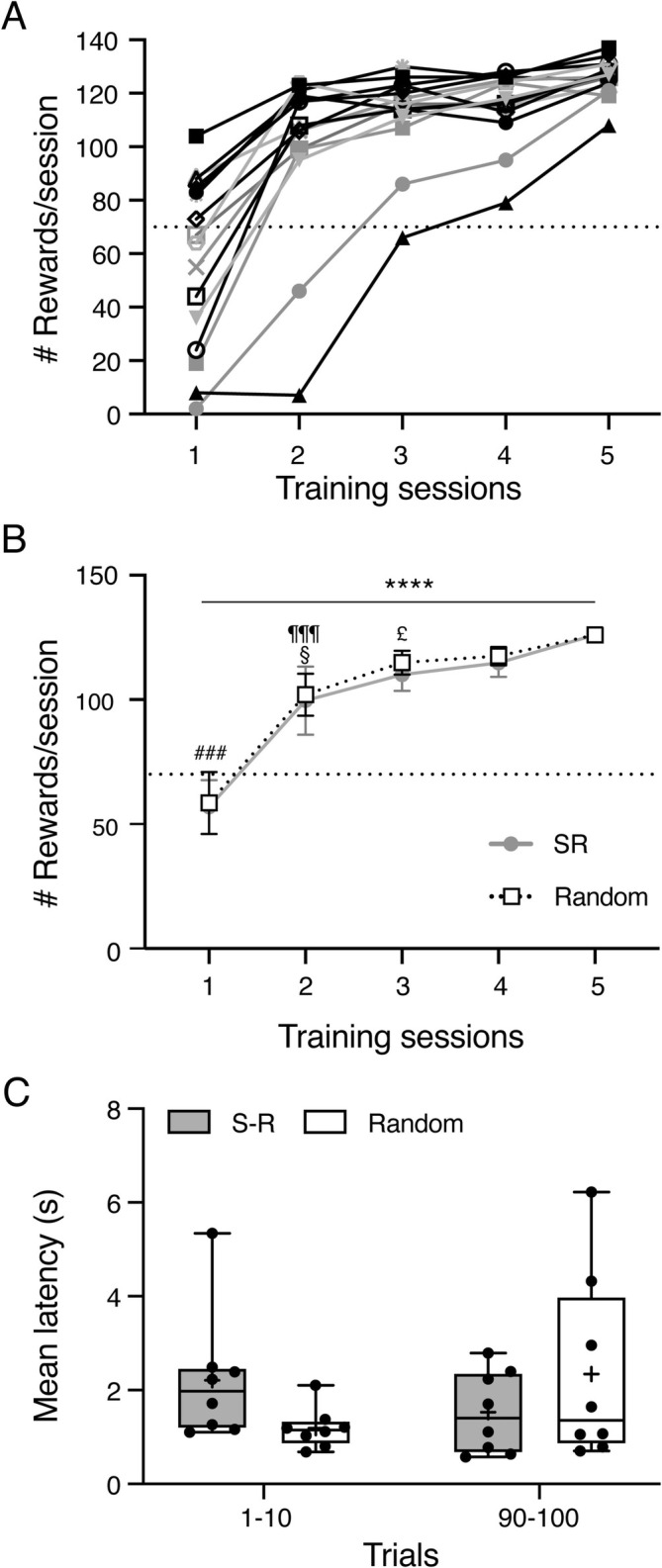
Behavioral performance in the main experiment. (A) Pre‐training. Graph shows the number of retrieved rewards per session for each rat in all pre‐training sessions. Dotted horizontal line indicates the 70 trials per session threshold defined as successful learning of the food‐retrieval aspect of the task. (B) Graph shows mean ± SEM of data in (A), split by group (SR (signaled reward), gray circles and line; random, white squares and dashed line. Solid horizontal line and **** indicate *p* < 0.0001 for the main effect of *Training Session* (two‐way ANOVA). ^###^ *p* < 0.001 compared to each other session 2–5; §, *p* < 0.05 compared to session 4; ^¶¶¶^ *p* < 0.001 compared to session 5; ^£^ *p* < 0.05 compared to session 5, Holm‐Šidák post hoc tests. (C) Box and whiskers plot shows all data points (black dots), median (horizontal line) and mean (+ symbol) of first nose poke latencies for the first and last 10 trials of the experimental session for S‐R (gray) and random (white) groups. Box shows 25th to 75th percentiles, and whiskers mark minimum and maximum values.

Figure 3A shows the development of food retrieval in the behavioral environment over the course of the pre‐training sessions. Most rats (14/16) reached the threshold for successful learning of the food‐retrieval aspect of the task (> 70 trials per session) after two sessions, with many already at that level within session 1, and all rats sustained criterion performance for at least 2 consecutive days by day 5; indeed, on day 5, all 16 rats obtained more than 100 reward deliveries. Figure 3B shows the mean ± SEM number of rewards per session separately for animals that were subsequently allocated to S‐R or random groups. The groups were extremely similar, and two‐way ANOVA confirmed a significant main effect of *Training Session* (F_(1.909, 26.72)_ = 49.05, *p* < 0.0001) reflecting the learning effect, with no effect of *Group* (F_(1, 14)_ = 0.064, *p* = 0.8) and no significant interaction (F_(4,56)_ = 0.054, *p* = 0.99). Post hoc testing (Holm‐Šidák) using data pooled across groups revealed that all sessions from 2 to 5 were significantly higher than session 1 (all *p* < 0.001), session 4 significantly higher than session 2 (*p* = 0.023), and session 5 significantly higher than both session 2 (*p* < 0.001) and session 3 (*p* = 0.046).

Following the successful performance in the pre‐training, rats were randomly assigned into either the S‐R or random group (*n* = 8 each) for the experimental session. As expected, there was no difference in the pre‐training performance in the last session between the rats allocated in the two groups (S‐R, 126.0 ± 8.0 trials; random, 126.0 ± 5.6; *U* = 25.50, *p* = 0.520, Mann–Whitney test). Further, in the subsequent experimental session, all but two rats across both groups achieved close to the maximal possible number of reward (set to 100) within the 30‐min session, with again no difference between the groups (S‐R, 96.3 ± 3.3; random, 91.1 ± 16.8 rewards; *U* = 22.0, *p* = 0.271, Mann–Whitney test).

In the experimental session, the house‐light cue was introduced for the first time so was a novel stimulus for both rat groups but was predictive of food reward delivery in only the S‐R group. To assess whether this difference in contingency produced a detectable behavioral effect within the single training session we compared the average latencies of the first nose‐poke following dispenser activation for the first and last 10 trials of the session (Figure 3C). In one rat in the S‐R group, there was a single outlier trial among the last 10 trials during which the rat spent > 90 s grooming, with a resultant latency that was > 3 SD from the average (Grubbs' test *G* = 2.651), and > 4 SD from the average of all trials (Grubbs' test *G* = 8.054). This outlier trial was rejected from analysis. Repeated measures two‐way ANOVA for factors *Group* (S‐R, random) and *Time* (1st‐10, last‐10) revealed a *Group* × *Time* interaction term that approached but did not reach significance (F_(1, 14)_ = 3.932, *p* = 0.067). There were no main effects of *Group* (F_(1, 14)_ = 0.049, *p* = 0.827) or *Time* (F_(1, 14)_ = 0.257, *p* = 0.620).

#### 3.2.2 Retrograde Expression of GFP in PVT

**FIGURE 4:**
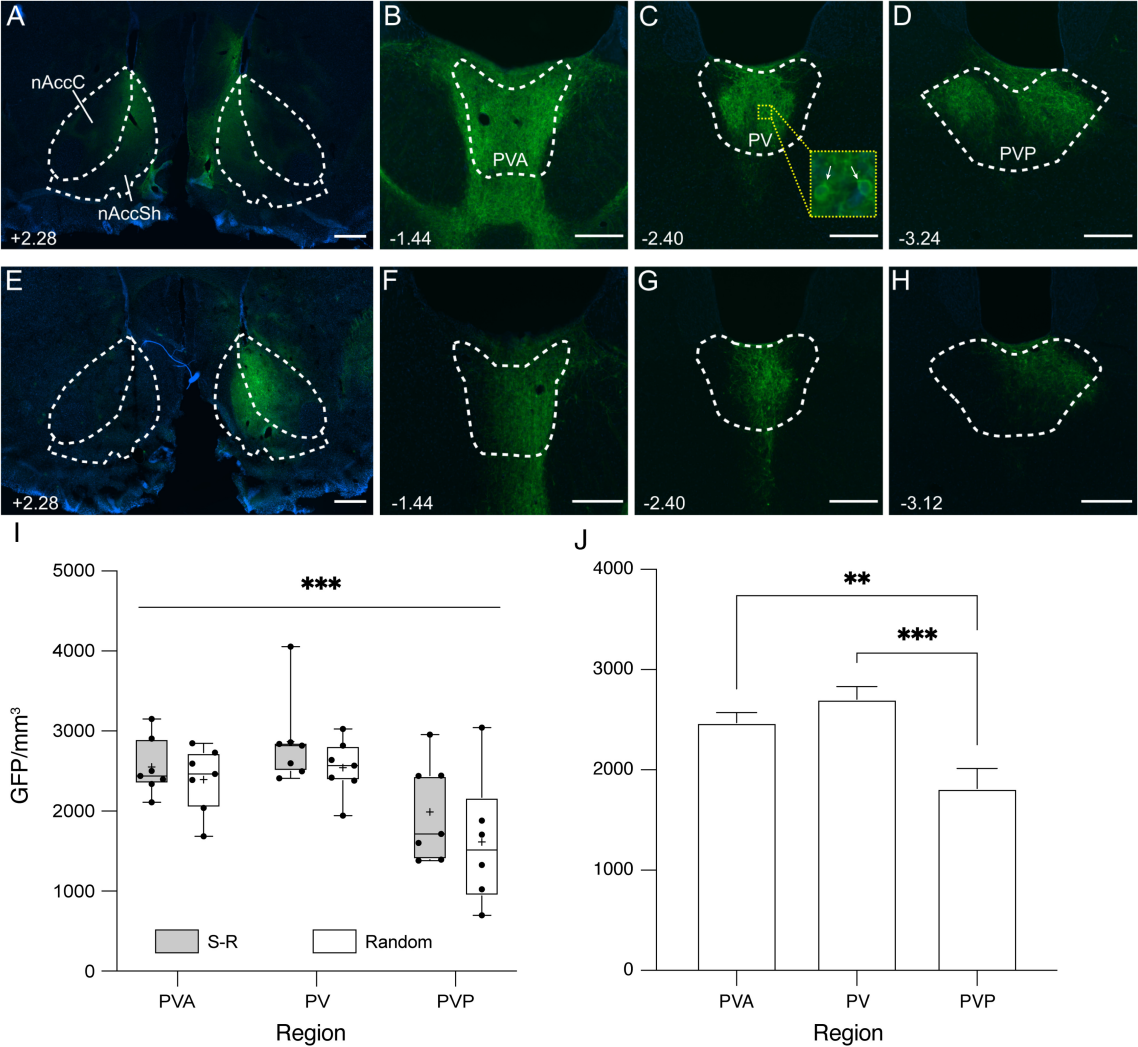
GFP‐expression in nAcc and PVT following virus injections in the nAcc. (A) Coronal section labelled for GFP (green) and DAPI (blue) from a rat showing GFP expression at the injection site in the nAcc in both hemispheres. The anteroposterior level of sections relative to bregma (Paxinos and Watson 2009) is indicated bottom left. Scale bar = 500 μm. nAccC, nucleus accumbens core; nAccSh, nucleus accumbens shell. B‐D. Representative sections from the same rat in (A) through each of the sub‐regions of the PVT (scale bars = 250 μm in each panel) showing bilateral retrograde labelling in (B) anterior (PVA), (C) mid (PV), and (D) posterior PVT (PVP). Inset in (C) shows enlarged region to illustrate GFP in cell bodies (arrows). (E) Coronal section from a rat in which injection was only successful on one side, showing GFP labelling limited to the right nAcc. Scale bar = 500 μm. (F–H) Sections from the same rat as in (E) show lateralized labelling in PVT sub‐regions (scale bars = 250 μm in each panel). (I) Box and whiskers plot shows all data points (black dots), median (horizontal line), and mean (+ symbol) density of GFP‐labelled cells for rats in S‐R (gray bar) and random (white bar) groups, split by PVT regions. Box shows 25th to 75th percentiles, and whiskers mark minimum and maximum values. Horizontal bar and *** indicate significant main effect of *Region* (*p* < 0.001, two‐way ANOVA). Post hoc analysis is shown in (J). There was no effect of *Group* or interaction. (J) Graph shows post hoc analysis of the main effect of *Region* on mean + SEM density of GFP labelled cells from (I), pooled across group. **p < 0.01, ***p < 0.001, Holm‐Šidák post hoc tests.

Rats in the main experiment received injections of AAVrg‐Syn‐ChR2(H134R)‐GFP aimed at the medial nAcc to retrogradely label projection neurons in PVT. In 14/16 rats the nAcc was successfully targeted bilaterally, with all the injection sites encompassing the dorsomedial and medial nAcc shell with a few cases also including ventromedial nAcc shell and part of the nAcc core. In these 14 rats, there was retrograde GFP labelling of neurons bilaterally throughout the rostro‐caudal extent of the PVT, as illustrated by the example shown in Figure 4A–D. At the level of PVA there was also GFP labelling in more ventral midline thalamic nuclei (e.g., Figure 4B). Of the two remaining rats (one from each group), the injections in one were too anterior and missed the nAcc target bilaterally; in the other, labelling was only observed on one side of the brain, in the right nAcc (Figure 4E). In this rat retrograde expression of GFP was seen mainly in the ipsilateral PVT (Figure 4F–H), consistent with previous findings that, although PVT spans the midline, outputs to nAcc have a lateralized aspect (Li and Kirouac 2008; Kirouac 2015). Both these rats were excluded from GFP and double‐label analyses (leaving *n* = 7 rats per group), to optimize uniformity of numbers of PVT projection neurons between the groups. However, because c‐Fos expression was presumed independent of GFP labelling, all rats (*n* = 8 in each group) were used for analysis of c‐Fos expression alone.

For the 14 rats included in analyses of GFP, the average number of GFP labelled neurons per 40 μm section across the entire PVT was 25, with averages of 28, 23, and 22 for PVA, PV, and PVP, respectively, and the average total number per rat was 467. To normalize for variations in volume of PVT across subregions and between rats, for statistical comparisons, the volume of the regions included within PVT boundaries was quantified and raw counts converted to density. As a necessary control for the subsequent comparison of GFP and c‐Fos double labelling between the two groups of rats, we first confirmed that they had equivalent density of GFP labelling in PVT. The data are shown in Figure 4I. Two‐way ANOVA for *Group* (S‐R, random) and *Region* (PVA, PV, PVP) confirmed no *Group* × *Region* interaction (F_(2, 35)_ = 0.159, *p* = 0.854), or main effect of *Group* (F_(1, 35)_ = 2.938, *p* = 0.095), as expected in these randomly allocated groups. Overall, there were on average 2.183 ± 0.5 × 10^3^ GFP‐labelled cells per mm^3^ in random and 2.47 ± 0.444 x 10^3^ per mm^3^ in S‐R group rats. However, there was a significant main effect of *Region* (F_(2, 35)_ = 10.24, *p* < 0.001), with post hoc comparisons (Holm‐Šidák; Figure 4J) confirming lower density of retrogradely labeled neurons in PVP (1.816 ± 0.716 × 10^3^ per mm^3^) compared to both PV (2.706 ± 0.475 × 10^3^; *p* < 0.001) and PVA (2.472 ± 0.377 × 10^3^; *p* = 0.005), and no difference between PVA and PV. Our injections of the retrogradely transported marker predominantly affected the dorsal nAcc shell, and so this regional effect is consistent with the previous observation that anterior PVT tends to project more dorsally in NAcc, whereas posterior neurons project more ventrally (Dong et al. 2017).

#### 3.2.3 Higher c‐Fos Activation in S‐R Than Random Group Animals

**FIGURE 5:**
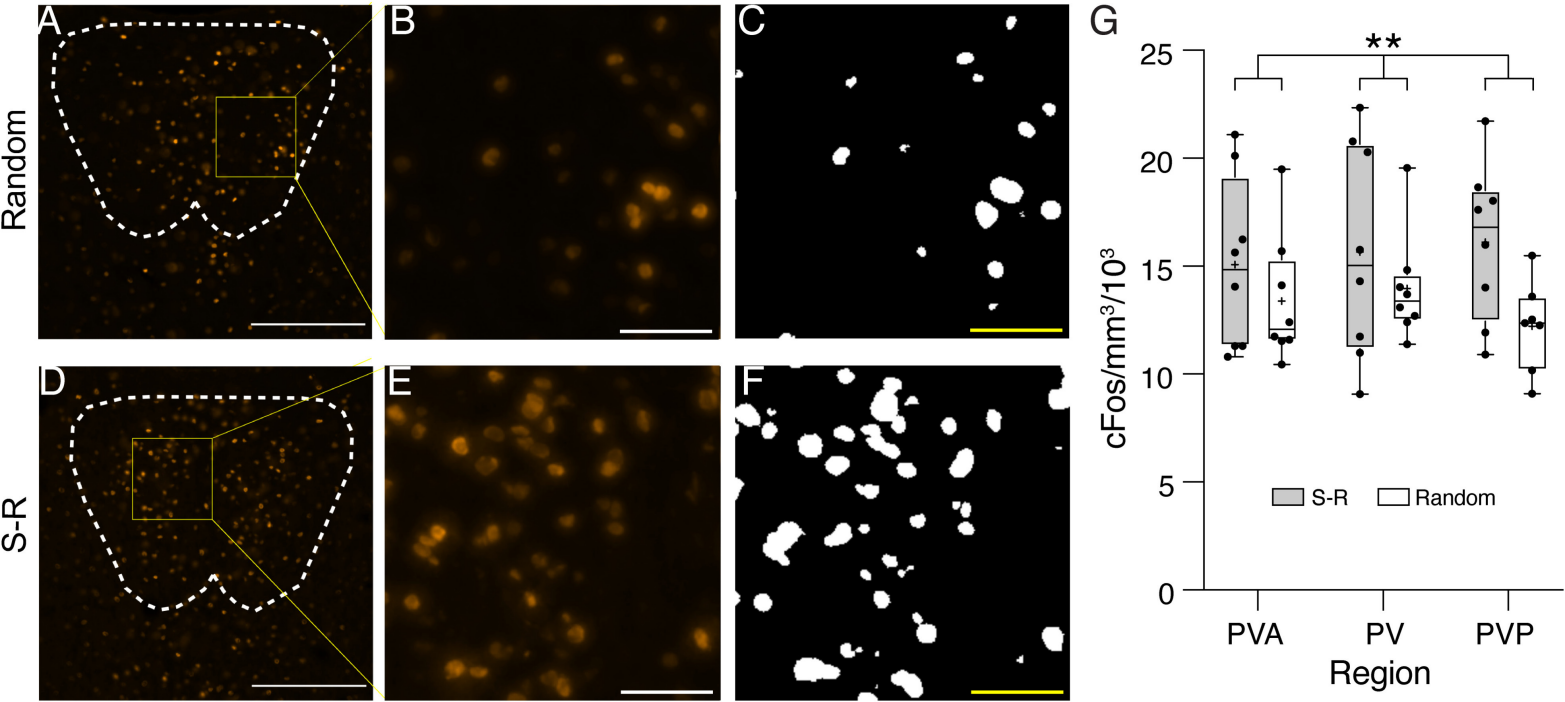
Enhanced c‐Fos activation in PVT following exposure to visual stimuli paired with reward. (A) Image shows a PVT section from a typical random group rat, at AP − 2.52 mm from bregma (Paxinos and Watson 2009) stained for c‐Fos (orange). Scale bar = 250 μm. (B) Enlargement of the region in box in (A). Scale bar = 50 μm. (C) Image shows the output of the ImageJ Particle Analysis selection channel, indicating (white) nuclei assessed as c‐Fos positive for the region in (B). Scale bar (yellow, to separate from selection output) = 50 μm. (D) A PVT section at the same anteroposterior coordinate as (A), from a typical S‐R group rat. Scale bar = 250 μm. (E) Zoom in on box in (D). Scale bar = 50 μm. (F) Output of the selection channel for the region in (E). Scale bar (yellow) = 50 μm. (G) Box and whiskers plot shows all data points (black dots), median (horizontal line), and mean (+ symbol) density of c‐Fos‐labelled nuclei (in thousands/mm^3^) for S‐R (gray bars) and random (white bars) groups, split by PVT region. Box shows 25th to 75th percentiles, and whiskers mark minimum and maximum values. **p < 0.01 main effect of *Group*, two‐way ANOVA.

We next analyzed the effect of pairing the cue and reward delivery on c‐Fos activation. On the basis of our previous study (Igelstrom et al. 2010), we expected higher density in the S‐R compared to the random group. For this, we were able to use all animals that performed the experimental session (*n* = 8 each group). This analysis revealed that indeed the density of c‐Fos expression was higher in the S‐R group. Histology from a typical random‐group animal is shown in Figure 5A–C, and from an S‐R‐group animal in Figure 5D–F. The quantitative analysis of c‐Fos expression densities is shown in Figure 5G. A two‐way ANOVA for factors *Group* (S‐R, random) and *Region* (PVA, PV, PVP) confirmed that the S‐R group showed higher density of c‐Fos activation in PVT (15.609 ± 0.522 × 10^3^ nuclei per mm^3^) than the random group (13.183 ± 0.889 × 10^3^ per mm^3^; main effect of *group* F_(1, 41)_ = 5.533, *p* = 0.024). There was no significant main effect of *Region* (F_(2, 41)_ = 0.162, *p* = 0.851) and no interaction (F_(2, 41)_ = 0.494, *p* = 0.614). In addition, we compared the c‐Fos densities for S‐R group animals in this main experiment with the animals exposed to either cues or rewards in the baseline study, pooled across regions. An unpaired *t* test confirmed that the mean in the S‐R group, in which cues predicted rewards (15.6 ± 0.522 × 10^3^ nuclei per mm^3^), was higher than the mean for the baseline animals exposed to one or other of these stimuli alone (14.09 ± 0.516 × 10^3^ nuclei per mm^3^; *t*_(4)_ = 3.59, *p* = 0.02). Together, these data confirm that c‐Fos activation was enhanced following a single session of exposure to reward‐predicting cues during S‐R conditioning, and that this occurred to equal extent across the rostro‐caudal extent of PVT. These results replicate and extend the findings of our previous study, which was limited to counts/mm^2^ within 30‐μm‐thick sections at a single anteroposterior level of PVT (Igelstrom et al. 2010).

As an important control we considered whether the difference we found between S‐R and random groups in the main experiment could be related to differences between the groups in the number of rewards obtained. However, arguing against this, as noted in the behavioral analysis above, all but two rats across both groups achieved close to maximal possible trial numbers in the session (100 trials). Further, the c‐Fos densities in PVT did not correlate with the number of reward trials achieved in the conditioning session overall in either group (S‐R: *r*_*s*_ = −0.108, *p* = 0.806; Random: *r*_*s*_ = −0.218, *p* = 0.607, Spearman correlation). The two rats with lower trial numbers (58 and 71 trials) were both in the random group; however, even in these two rats there was no obvious correlation, with one having among the lowest and the other among the highest c‐Fos densities observed. These data strongly suggest that reward number was not a main contributor to group differences in c‐Fos density.

Finally, while the behavioral analysis reported above (Figure 3C) did not find a significant reduction in the mean nose‐poke latency in the S‐R group, we checked if changes in behavior during the S‐R conditioning session at the individual animal level might relate to the level of c‐Fos density seen in those animals. For this, we calculated the Spearman correlation between the change in nose poke latency from the first to last 10 trials and the average c‐Fos density across the entire PVT for S‐R group rats (*n* = 8). However, there was no significant correlation (r = 0.1429, *p* = 0.75).

#### 3.2.4 Enhanced c‐Fos Activation With S‐R Conditioning Occurs in PVT Neurons Projecting to nAcc

**FIGURE 6:**
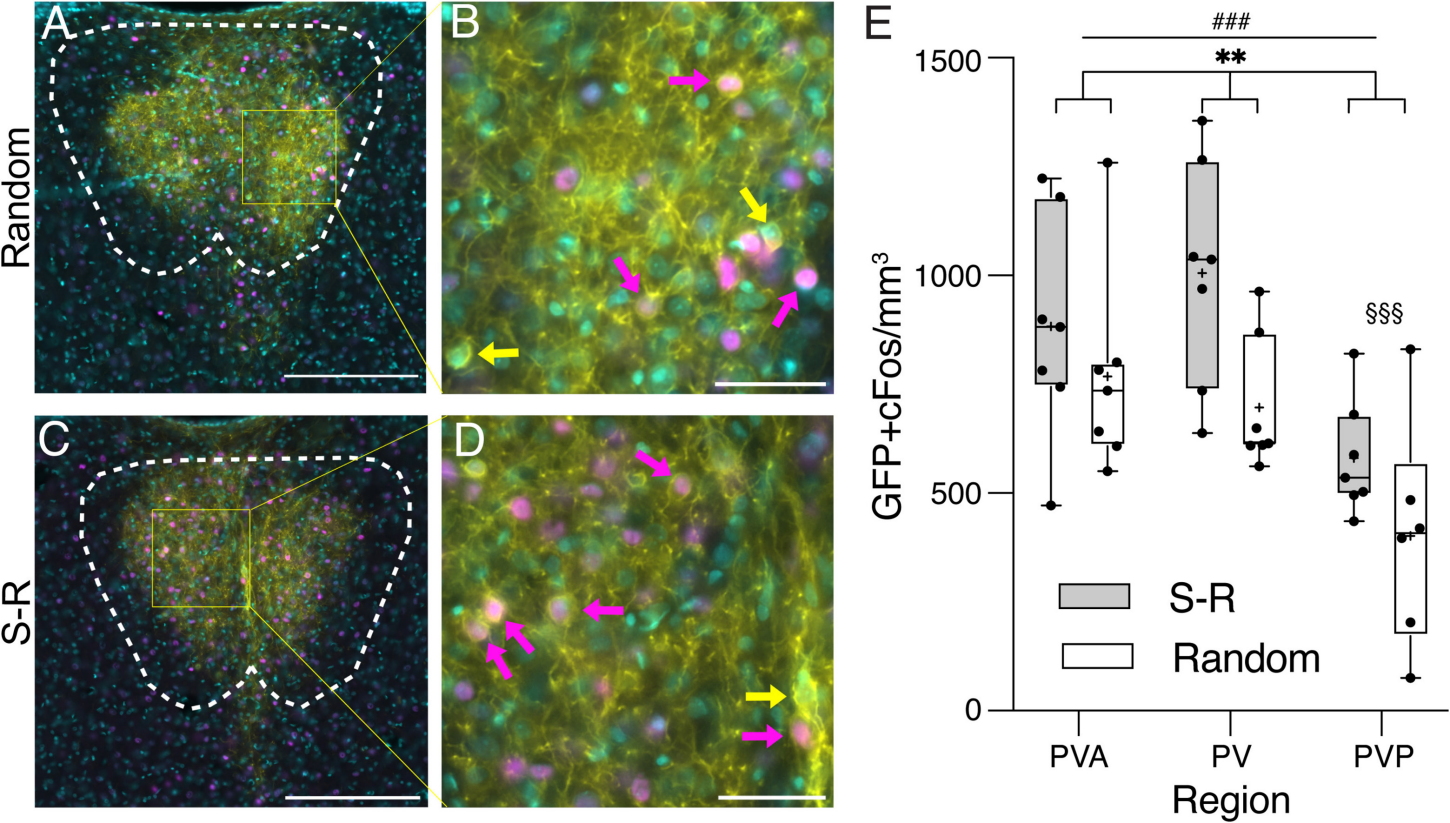
c‐Fos activation in PVT neurons projecting to nAcc. (A) Example image of a PVT section at AP ‐2.52 mm from bregma (Paxinos and Watson 2009) from a random group rat. To improve contrast of overlapping color regions in the three‐color images in this figure, the GFP channel was set to yellow, c‐Fos to magenta, and DAPI to cyan. Thus c‐Fos positive nuclei in GFP positive neurons show as pink surrounded by yellow cytoplasm/membrane, whereas c‐Fos negative nuclei in GFP positive neurons show as light green surrounded by yellow. Scale bar = 250 μm. (B) Enlargement of the region in box in (A). Magenta arrows indicate examples of neurons co‐labelled with c‐Fos and GFP, yellow arrows indicate neurons only labelled with GFP. Scale bar = 50 μm. (C) Example image of a PVT section from a S‐R group rat. Scale bar = 250 μm. (D) Enlargement of region in (C). Scale bar = 50 μm. (E) Box and whiskers plot shows all data points (black dots), median (horizontal line), and mean (+ symbol) density of cells double‐labelled with GFP and c‐Fos. Box shows 25th to 75th percentiles, and whiskers mark minimum and maximum values, for S‐R (gray bar) and random (white bar) groups split by PVT region. **p < 0.01 for main effect of G*roup,* ^###^ *p* < 0.001 for main effect of *Region* (two‐way ANOVA), ^§§§^ *p* < 0.001 for PVP compared to both PVA and PV, Holm‐Šidák post hoc tests.

The primary goal of this study was to determine if the enhanced c‐Fos activation in the S‐R condition observed previously and repeated here occurs in neurons projecting to nAcc. To address this question, we quantified the expression of GFP and c‐Fos double labelling in neurons within the boundaries of PVT. For this analysis, we only used animals which had successful bilateral injections of AAV in nAcc, leaving *n* = 7 in each group. Representative histological sections from one typical rat from each group are shown in Figure 6A–D. While double‐labelled cells are seen in both rats, there are fewer in the random group animal (Figure 6A,B) than in the S‐R group animal (Figure 6C,D). This was confirmed by the quantitative analysis shown in Figure 6E, with two‐way ANOVA revealing a significant main effect of *Group* (F_(1, 35)_ = 8.387, *p* = 0.006) on the overall density of double‐labelled neurons, with a higher density of double‐labelled neurons in rats exposed to the S‐R condition (823 ± 219 double‐labelled cells per mm^3^) than in the random group (622 ± 194 per mm^3^).

There was also a significant effect of *Region* (F_(2, 35)_ = 10.92, *p* < 0.001) with significantly lower density of double‐labelled cells in PVP (491 ± 126 per mm^3^) than both PV (852 ± 219, *p* < 0.001, Holm‐Šidák post hoc test) and PVA (826 ± 81, *p* < 0.001), with no difference between PVA and PV (*p* = 0.761). Importantly however, there was no *Group* × *Region* interaction (F_(2, 35)_ = 0.70, *p* = 0.504), indicating that the enhancement of c‐Fos expression in projection neurons in S‐R compared to random group animals occurred across the entire anteroposterior extent of PVT. The reduced density of double‐labelled neurons in PVP compared to other PVT regions is likely simply due to the reduced density of GFP labelled neurons in PVP, described above (Figure 4). To test this hypothesis and fully rule out the possibility that some between‐group differences in uptake of the retrograde tracer may have affected the number of double‐labelled cells found, we analyzed the ratio of GFP + cFos double‐labelled neurons to total GFP labelled neurons. A two‐way ANOVA again confirmed a significant main effect of *Group*, with a higher proportion (34%) of GFP labelled neurons expressing cFos in the S‐R group (average ratio 0.336 ± 0.026) than the Random group animals (28%; average ratio 0.278 ± 0.044; F_(1,35)_ = 5.1, *p* = 0.03). For these proportion data, there was no significant main effect of *Region* and no significant *Group × Region* interaction, confirming that the effect of *Region* for absolute densities of double‐labelled cells noted above was due to the reduced density of GFP labelled projection neurons in PVP compared to the other subregions. Thus, compared to random group animals in which cues were not predictive of reward, a single session of cue‐reward pairing in the S‐R group significantly enhanced c‐Fos expression in the specific subset of PVT neurons with projections to nAcc, as it does for the population overall, and this applies equally to projection neurons across all PVT subregions.

To determine if the increase in c‐Fos expression seen in S‐R group animals was more pronounced in GFP‐expressing (projection) neurons than for non‐GFP‐labelled neurons, we performed a contingency analysis of the ratio of the density of GFP + cFos double‐labelled neurons to the total c‐Fos neuron density in S‐R and random group animals. The total c‐Fos densities were 15,609/mm^3^ and 13,226/mm^3^ for S‐R and random group animals, respectively, and the average double‐labelled densities were 823/mm^3^ in S‐R and 633/mm^3^ in the random group. Expressed as percentages, the respective ratios of double‐labelled c‐Fos density to total c‐Fos density were 5.27% in S‐R and 4.79% in random group rats. With the numbers available the analysis approached but did not reach significance (*p* = 0.075, Fisher's exact test). However, these data need to be interpreted cautiously because as noted above, non‐GFP‐labelled neurons may include projection neurons not labelled by the tracer injections.

## 4 Discussion

In the present study, we first confirmed our previous results showing that following a single session of pairing a novel sensory cue with food reward, there is increased c‐Fos expression in PVT neurons compared to un‐paired control animals. Further, we extended that study by showing that this effect is consistent across the anteroposterior extent of PVT. The main new finding of the study was to show that the subpopulation of PVT neurons that project to nAcc share the enhanced expression of c‐Fos following cue‐reward pairing seen in the general population.

The results showing enhanced c‐Fos activation in PVT in cue‐reward paired animals are concordant with several previous studies in trained animals (Brown et al. 1992; Dayas et al. 2007; Dayas et al. 2008; Choi et al. 2010; Flagel et al. 2011; Yager et al. 2015) and confirm our previous findings for animals in early stages of learning (Igelstrom et al. 2010). Here, we repeated many of the critical features of the experimental design used in our previous study, such as the experimental environment, animal handling and training, the nature of the sensory cue, cue duration, cue‐reward interval, number of trials, duration of the session, and post session delay before sacrifice for c‐Fos staining. However, there were some differences in the designs. For example, whereas the previous study utilized a liquid reward with non‐calorific sweetener, the present one employed a nutritionally balanced but flavored solid food. Consequently, the required food retrieval behaviors also changed, from licking at a spout to nose entry to a feeder. Thus, the present results enable the original observation to be generalized to a wider range of situations.

One of the factors held constant between this and our previous study was the use of only male rats, with this gender bias also present in all the previous studies of PVT neuronal activation under reward learning contexts cited here, both above for the general population and below for neurons with projections to the nAcc. A recent study that investigated activation of the PVT to amygdala projection neurons in response to novelty found no difference between male and female rats (Greiner and Petrovich 2024). Nevertheless, potential sex differences in PVT activation during cue‐reward conditioning cannot be ruled out, and this should be addressed by future studies.

Another aspect of the shared design was a relatively short interval from the end of the training session until the sacrifice of the animal. This meant that the c‐Fos changes seen were very likely associated with trials quite early in the training session. It is possible that choosing a longer delay until sacrifice might lead to an enhanced differential c‐Fos signal between the groups because learning might be expected to be further developed later in the session. However, the fact that we were able to detect a significant difference even at this early stage suggests that something about the existence of a temporal association between the cue and the reward delivery was already being signalled to PVT neurons, a vital step towards developing a conditioned response to the cue.

The finding of c‐Fos activation in cue‐reward paired animals is consistent with results using other methods to monitor neural responses. In single neuron recording experiments we (Li et al. 2016) and others (Zhu et al. 2018; Meffre et al. 2019; Munkhzaya et al. 2020) have found neurophysiological activation to be a common response to reward‐predicting cues in PVT of well‐trained animals, and calcium signals reflecting underlying physiological activation occur in some PVT neurons in response to reward‐predicting cues during learning of associations (Zhu et al. 2018; Choi et al. 2019). The expression of c‐Fos may indeed be triggered by neurophysiological activation of the neurons via calcium influx (Sheng and Greenberg 1990). However, it is unclear across these studies whether the neurophysiological responses show the level of differential activation that could account for that seen in the c‐Fos data.

Despite the differential c‐Fos expression, measures of conditioned behavior in response to the cue revealed no differential effects between groups in either this or our previous study, whereas successful learning of the cue‐reward association might be expected to lead to increased anticipatory behavior following the cue. There are several possible reasons why behavioral signs of learning might not have been detected. One is that there may be a floor effect in the measure used (latency to first nose‐poke) that makes it hard to demonstrate significant changes. At a functional level, another possibility is that the development of the behavioral expression of learning as measured in these studies lags the development of physiological changes in brain circuits, and the behavior had not reached threshold for detection within the single session. For example, mice in which PVT activity was studied in a similar conditioning task only developed behavioral responses to the cue after 4 days of training (Otis et al. 2019). Importantly, this demonstrates that given time, rodents do develop measurable behavioral responses in contexts like those employed here. A third possibility is that the population of rats included both goal‐ and sign‐tracking animals. Whereas goal‐trackers respond following paired associative learning by approaching the site of predicted reward delivery, sign‐trackers attend the cue rather than the reward site (Haight and Flagel 2014), so their presence might have obscured differences in our behavioral measure at the population level. Indeed, previous studies suggest that it might be sign‐trackers in particular that show differential activation in PVT by reward‐associated cues (Flagel et al. 2011; Yager et al. 2015), raising the possibility that the results seen in our two studies arise predominantly from such a subset of rats in each case. Further work is required to explore this possibility. Interestingly, even if sign‐ versus goal‐tracking tendencies affected the ability to detect behavioral changes, there is nevertheless a temporal aspect to that factor; it has been found that sign‐tracking behavior also takes time to develop, only becoming evident after two training sessions (Flagel et al. 2011), i.e., beyond the period available to the rats in the present experiment. Therefore, irrespective of whether the animals studied here would have developed sign‐ or goal‐tracking behavior, the findings remain consistent with the idea that the c‐Fos changes reflect neural changes occurring early in learning, preceding the time at which observable behavioral expression is generally found.

A question remaining from our previous study was whether the observed effect was specific to the mid‐region of PVT, which was the only anteroposterior level examined by Igelstrom et al. (2010). This is potentially important, because, as recently reviewed by Barson et al. (2020), there are gradations in input–output connectivity and in the specific functional correlates between anterior and posterior regions of PVT. However, our quantitative analysis of anterior, mid, and posterior zones of PVT showed no differences between the regions in the level of c‐Fos activation in response to different behavioral situations. This was the case both in the baseline study, where there were no differences between regions in expression of c‐Fos across animals exposed to different combinations of the test environment, and in the main study, where enhanced c‐Fos in animals exposed to cue–reward pairing versus un‐paired animals occurred equally across all three regions.

This regional uniformity in c‐Fos expression contrasts with some previous studies which have found regional differences in activation, using a variety of methods to quantify activity, in a range of different behavioral paradigms. Multiple factors can contribute to apparent inconsistencies in findings between studies, such as differential neonatal experiences during the life history (Kooiker et al. 2021) or, more directly, sampling of different neuronal populations, or variations in behavioral paradigms affecting variables such as arousal or metabolic state at the time of the experiment (De Groote and de Kerchove d'Exaerde 2021). The sensitivity and temporal resolution of the recording methods may also be important. For instance, a recent calcium imaging study of PVT activation in animals during learning of a cue‐reward association found differential responding between anterior and posterior regions to reward consumption (Choi et al. 2019), whereas here we saw no regional difference in the baseline study between chamber only and chamber plus food‐reward exposed animals. Calcium imaging has high temporal resolution and can analyze responses to specific intra‐block effects, while c‐Fos imaging as employed here reveals the net combined effect on gene expression of all stimuli occurring during the trial block. Indeed, Choi et al. (2019) found no difference between regions in the response to the cue, and brief phasic responses may differ in the extent to which they are associated with differential gene expression.

Variation between studies in the stage of learning at the time of testing is another potential factor. For instance, whereas here we studied responses very early in learning a cue‐reward association, Haight et al. (2017) examined c‐Fos expression in previously well‐trained animals, following a session in which cues but not rewards were delivered, in a different environment to that in which animals first learned the cue‐reward association. They found a significant difference between sign‐ and goal‐tracking rats but only for posterior but not anterior or mid PVT, and the results were interpreted as suggesting posterior PVT has a particular role in incentive salience. Conversely, another study under extinction‐like conditions found that pharmacological inactivation of anterior but not posterior PVT impacted reward‐seeking behavior when expected reward is omitted (Do‐Monte et al. 2017). Further work is required to unravel the implications of the findings of potential differential functional roles of anterior and posterior PVT, when examined under extinction‐like conditions, on the interpretation of the uniformity of c‐Fos activation we found during early learning.

The main goal of the present study was to examine c‐Fos expression specifically in PVT neurons projecting to nAcc following a single session of cue‐reward pairing, i.e., very early in associative learning. Our analysis of the density of neurons double labelled for GFP and c‐Fos supported the hypothesis that the subset of PVT neurons that project to nAcc is more activated in animals exposed to cue‐reward paring than in the group in which cues and rewards were not linked. Projections to nAcc form the largest proportion of efferents from PVT (Su and Bentivoglio 1990; Moga et al. 1995; Pinto et al. 2003; Dong et al. 2017), and are therefore of key importance given the central role of nAcc in regulation of reward‐related behavior (Ikemoto and Panksepp 1999). Neurons with projections to nAcc are of particular importance not only for directly affecting nAcc medium spiny neuron excitability via terminals on dendritic spines (Pinto et al. 2003; Ligorio et al. 2009), but also for modulating dopaminergic transmission in the nucleus (Jones et al. 1989; Parsons et al. 2007). Dopamine is a key player in enabling synaptic plasticity for learning (Reynolds et al. 2001). Furthermore, enhanced expression of c‐Fos is itself implicated in synaptic plasticity mechanisms for learning and memory (Tischmeyer and Grimm 1999; West et al. 2002).

Here, we used a retrogradely transported AAV‐ChR2‐GFP construct to label PVT to nAcc projection neurons through GFP expression. Such constructs are commonly used for functional studies of specific pathways using optogenetic stimulation, on the assumption that the construct reliably retrogradely labels the neurons of origin of the pathway. However, purely anatomical studies usually employ a simpler construct, lacking the channel rhodopsin component. It is uncertain whether results from the two constructs differ. We are not aware of any quantitative studies of PVT to nAcc projections using a non‐ChR2 AAV construct to compare our results with. Compared to previous quantitative studies of the PVT to nAcc projection using a different tracer, cholera toxin B, the raw numbers of GFP labeled cells we observed seem low. For instance, back calculating from the proportion data provided by Dong et al. (2017) indicates total numbers of projection neurons in the thousands, rather than the hundreds we found, and Li and Kirouac (2008) found 100 or more per 50 μm section, compared to around 25 per 40 μm section in the present study. Cholera toxin subunit B is a highly sensitive retrograde tracer that produces intense labeling from small injection sites (Conte et al. 2009). There are many other potential contributing factors apart from the properties of the tracer that may affect the number of projection neurons found, including the extent to which the tracer injection involved the nAcc. Thus, the densities we found may be an underestimate of the density of projection neurons in PVT. The low numbers may have contributed to the fact that, while a trend may be present, we were unable to detect a significant difference between GFP‐labeled and unlabeled cells in the proportion expressing c‐Fos. Importantly, however, we used the same methodology and mingled animals from both groups during processing, and for the present study the relative density of double labeled neurons in the two groups was the most important measure, rather than characterizing the absolute density of projection neurons per se.

The key novelty of the present findings is that we show enhanced c‐Fos expression in nAcc‐projecting PVT neurons very early in learning a cue‐reward association. This builds on previous reports of c‐Fos activation in PVT‐nAcc projection neurons in animals studied at later stages of the conditioning process. In well‐trained animals Haight et al. (2017) found enhanced c‐Fos in response to the previously conditioned cue in nAcc‐projecting PVT neurons compared to untrained control animals, albeit only in sign‐trackers. Interestingly, in the well‐trained animals the effect was only seen in posterior PVT, whereas here the whole nucleus was involved. That study was conducted during the light phase which, as noted above, could be a factor in contributing to differences between studies. It is also possible that there is some diminution in the overall activation following learning of the association, with posterior PVT remaining elevated only in sign trackers perhaps due to it having a particularly strong involvement in assigning incentive salience to cues in animals that develop sign tracking behavior (Haight et al. 2017). In another prior study, like the present one conducted during the animal's dark phase, nAcc‐projecting PVT neurons showed enhanced c‐Fos in animals expressing reinstatement of conditioned behavior following completion of extinction procedures (Hamlin et al. 2009).

An interesting contrast with the present findings was that in both earlier studies c‐Fos was only elevated in the projection neurons, and not in the population overall. This would also be consistent with a lesser PVT c‐Fos activation post‐learning phases compared to during initial learning. However, arguing against this, in other studies of well‐trained animals, conducted during the light phase, reward cues have been found to trigger increases in c‐Fos in the general PVT neuronal population (Flagel et al. 2011; Yager et al. 2015), as seen in the present study early in learning. Given these disparate results, further studies are required to clarify whether projection neurons are differentially activated compared to the general population at different stages of learning.

In contrast to the finding of increased c‐Fos activation in PVT projection neurons across multiple studies, a previous study in mice that used two‐photon calcium imaging to monitor the activity of individual nAcc‐projecting PVT neurons found that many developed *decreased* calcium signals, while a smaller number developed an increase during the learning of cue‐reward associations (Otis et al. 2019). As noted above, it remains unclear how such phasic, event‐related changes among different populations might relate to levels of c‐Fos expression visualized by immunohistochemistry. For instance, if calcium flux declined in cells that already had low levels of c‐Fos, a positive effect on c‐Fos in a smaller number of cells with enhanced calcium entry might dominate in a c‐Fos study. Other factors that may have affected results include potential differences between mice and rats (e.g., Hok et al. 2016), and testing during the animals' light phase, when mice and rats are less active (Refinetti 2006), compared to during the dark phase as in the present study. Whatever the case, the calcium imaging and c‐Fos data both lend support to the concept of dynamic physiological changes occurring in PVT projections to the nAcc during the learning of cue‐reward associations.

In conclusion, the findings of the present and prior studies taken together indicate that PVT neurons with projections to the nAcc show enhanced c‐Fos expression to reward‐associated cues across multiple phases of learning, extending from first exposure to cue‐reward association (the present study) to post‐extinction reinstatement of previously learnt associations. Plasticity in reward‐learning circuitry is likely to be required during all these phases. Thus, it will be of interest in future studies to investigate whether the differential c‐Fos changes seen between paired and unpaired cue‐reward contingencies of projections from PVT to nAcc reflect nuclear processes associated with activation of synaptic plasticity mechanisms at terminals of PVT projections in nAcc, related to initial stages of learning the association of the sensory cue with subsequent reward delivery.

## Author Contributions

**Sonja Seeger‐Armbruster:** conceptualization, methodology, formal analysis, data curation, writing – review and editing, visualization, supervision. **Mandy Wang:** formal analysis, investigation, writing – original draft, review and editing, visualization. **Rebecca Campbell:** methodology, resources, writing – review and editing, supervision, funding acquisition. **Brian Hyland:** conceptualization, methodology, formal analysis, writing – original draft, review and editing, visualization, supervision, project administration, funding acquisition.

## Conflicts of Interest

The authors declare no conflicts of interest.

## Peer Review

The peer review history for this article is available at https://www.webofscience.com/api/gateway/wos/peer‐review/10.1111/ejn.70168.

## Data Availability

The data that support the findings of this study are openly available in figshare at https://doi.org/10.6084/m9.figshare.29093480.
